# Supplementary figures and images for: Distinct expression requirements and rescue strategies for BEST1 loss- and gain-of-function mutations
Source: eLife. 2021 Jun 1;10:e67622. doi: 10.7554/eLife.67622 (PMC8169119; doi:10.7554/eLife.67622)

**Figure 2 ‒ source data 1. The uncropped blots in Figure 2d and Figure 3‒figure supplement 1.**


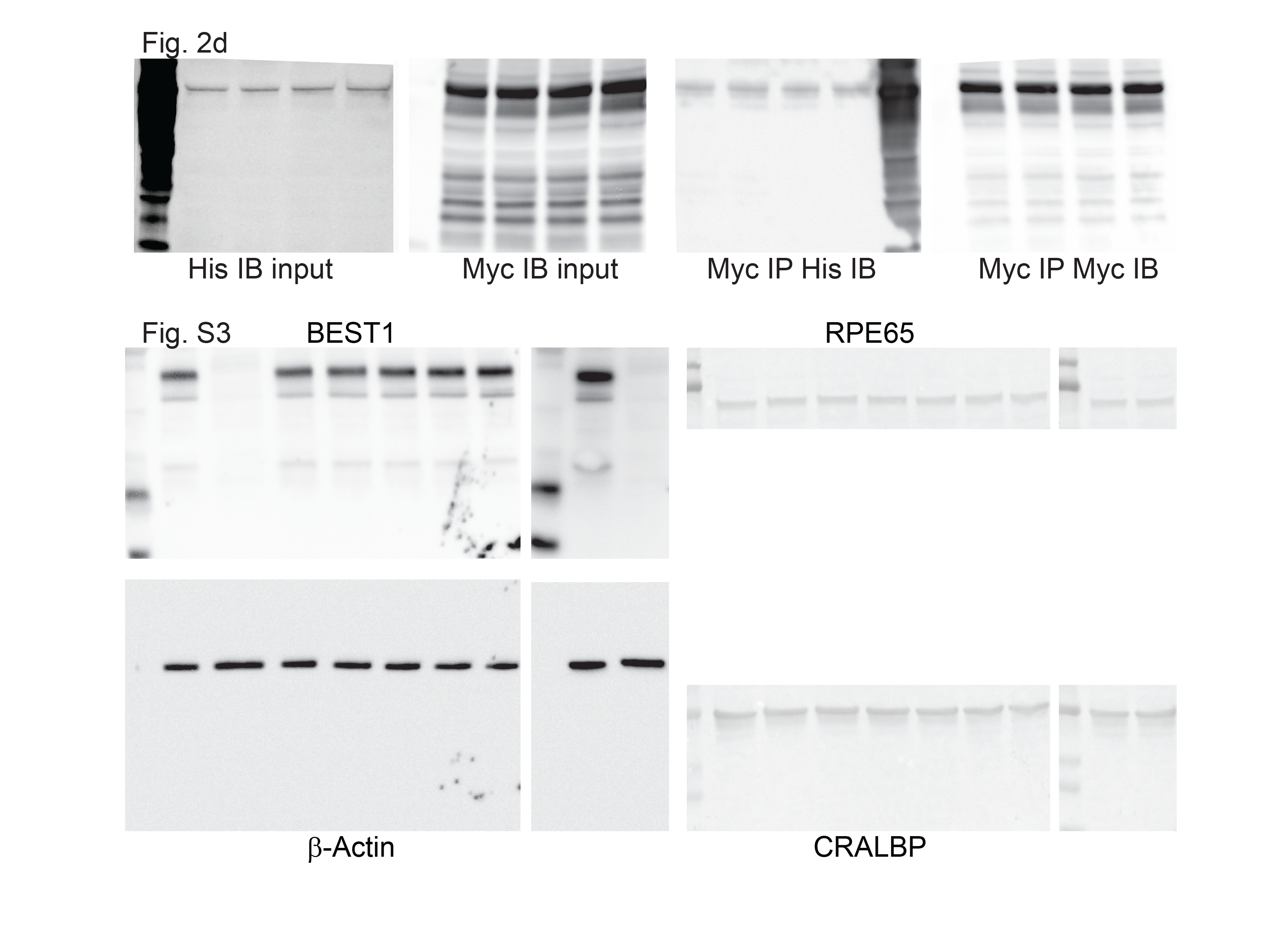

Supplement: Figure 2—source data 1. [file elife-67622-fig2-data1.docx]
