# Supplementary material for: Distinct expression requirements and rescue strategies for BEST1 loss- and gain-of-function mutations
Source: eLife. 2021 Jun 1;10:e67622. doi: 10.7554/eLife.67622 (PMC8169119; doi:10.7554/eLife.67622)
Supplement: Figure 3—source data 1. [file elife-67622-fig3-data1.docx]

**Figure 3‒source data 1. gRNA sequences for CRISPR/Cas9**

| Gene | Purpose | Targeting Exon | gRNA sequence |
| --- | --- | --- | --- |
| TMEM16A | knockout | Exon 10 | CCATGGTGGCGCATCCGTAC |
| TMEM16B | knockout | Exon 13 | GCTGGCCTGCGCGGTCCCAC |
| LRRC8A | knockout | Exon 3 | TCCTTGGTGACCCACTTACA |
| BEST1 | knockout | Exon 3 | CTCACCCAGCACGAAGGAAA |
|  | I205T knock-in | Exon 5 | TCTGGAGCAGGATAGGGTCC |
|  | Y236C knock-in | Exon 6 | TGTATACACAGGTGAGGACT |
|  | Silencing (BVSi 3-8) | Exon 3 | CTCACCCAGCACGAAGGAAA |
|  | Silencing (BVSi 5-4) | Exon 5 | GCAGGCTCTGGAGCAGGATA |
